# Response Surface Methods to Optimise Milling Parameters for Spirit Alcohol Production from Irish Wheat Grain

**DOI:** 10.3390/foods11081163

**Published:** 2022-04-16

**Authors:** Sinead Morris, John L. Byrne, Ben Murphy, Stephen J. Whelan, John P. Carroll, David Ryan

**Affiliations:** EnviroCORE, Department of Science and Health, Institute of Technology Carlow, R93 V960 Carlow, Ireland; john.byrne@itcarlow.ie (J.L.B.); c00242562@itcarlow.ie (B.M.); stephen.whelan@itcarlow.ie (S.J.W.); john.carroll@itcarlow.ie (J.P.C.); david.ryan@itcarlow.ie (D.R.)

**Keywords:** wheat, alcohol yield, response surface method, process optimisation

## Abstract

To standardise research activity and determine alcohol yield from native Irish hard wheat grain, a benchmark approach that reflects Irish industry norms is required. The goal of this study was to optimise milling parameters, grain particle size, and grain to liquid ratio towards developing a standard process. Hard wheat (*Triticum avestivum* cv. Costello) was used in this study. Experiments utilised a response surface method approach. When both 30 and 35 g of flour were used at a particle size of 0.2 mm, alcohol yield was >350 L of alcohol per tonne of grain (LA/tonne), but with a particle size of 0.65 and 1.1 mm, alcohol yield decreased to between 250 and 300 LA/tonne. It was noted that, during response surface study, >300 LA/tonne was achieved when grain amounts were >25 g, at a particle size of 0.2 mm; therefore, a follow-up experiment was conducted to determine whether there was a significant difference in grain amounts ranging from 25 to 35 g. During this experiment, no significant difference in alcohol yield was observed between 30 and 35 g of grain. Because there were no significant differences, the ideal milling parameters for alcohol yield were determined to be 30 g of flour with a particle size of 0.2 mm, achieving 389.5 LA/tonne. This study concludes that hard wheat can successfully be used for alcohol production, achieving >380 LA/tonne, when a milling size of 0.2 mm and more than 30 g of grain are used, and as such presents an opportunity for its increased use in Irish distilleries.

## 1. Introduction

Grain alcohol manufacturing in Ireland is heavily reliant on imported grains (non-GM maize grain), undermining the provenance of Irish spirits. The replacement of up to 100,000 tonnes of imported maize with Irish-grown grain has the potential to open up new markets for Irish farmers, while also allowing Irish whiskey to be made solely of native-grown grain. Annually, Ireland produces over 680,000 t of wheat grain, but it is unclear how much of that is used to make alcohol [1].

Wheat, both hard and soft, has been related to various processing difficulties in Irish distilleries, including foaming and high-viscosity residues, even though it is routinely used for alcohol production in other jurisdictions such as Scotland, with limited processing issues reported [2,3]. Foaming and viscosity can be particularly problematic due to the distinctiveness of the procedures used in Irish grain whiskey distilling, while also being attributed to the arabinoxylans and beta-gluten contents of the grain [4,5,6].

Because both agronomy and variety affect a grain’s potential to be converted into alcohol, it is unclear how well Irish cultivated grains would function in Irish grain whiskey manufacturing settings. Furthermore, the majority of Ireland’s wheat harvest is classed as hard wheat. Tillage farmers in Ireland commonly cultivate hard wheat, as it is well suited to both climate and environmental conditions, routinely yielding over 8 tonnes/ha [1]. However, the majority of Irish-grown wheat is sold as livestock feed, with a limited amount being used for bread and less still for grain alcohol production. Hard wheat has a higher protein content than soft wheat and distillers tend to dislike using it due to problems with increased viscosity and foaming during fermentation. Furthering this, there is an inverse relationship between protein and starch—as protein concentration within the grain increases, starch concentration decreases. Hardness has also been linked to processing issues, with the starch being less accessible and resulting in handling issues [7].

Previous studies and methodologies aimed at determining alcohol yield from wheat, using soft wheat, have focused on the Scotch whiskey production process [8,9]. However, as the Irish whiskey production process differs from that of Scotch [10,11], the results of this research cannot be directly extrapolated to the Irish context. For example, Scotch distilleries do not employ enzymes in grain alcohol production. According to the Irish whiskey technical file, “grain whiskey‘is produced from malted barley (not exceeding 30%) and includes whole un-malted cereals usually maize, wheat, or barley. Other natural enzymes may be used at the brewing and the fermentation stage” [10]. The addition of natural enzymes may allow for lower cooking temperatures and shorter mashing periods. This may be possible with natural enzymes such as α-amylase, amyloglucosidase, and β-glucanase, resulting in greater alcohol yield from wheat grain [12,13].

Currently, there is no lab scale process available, which mimics Irish industrial production norms, in place for testing smaller batches of wheat for potential processability issues and alcohol yield. This paper describes the development of a benchmark protocol, based on Irish industry norms, to determine alcohol yield from native Irish wheat grain, as part of creating an industry-standard procedure.

## 2. Materials and Methods

### 2.1. Cereal Sample and Composition

Wheat grain (cv. Costello) supplied by Goldcrop Ireland, was grown, and harvested in Ireland in 2019. *Costello* has a moisture content of 14%, protein of 11.25% and starch of 68.65%. Samples were stored in cool, dark conditions until required for use. Malted barley (cv. Laureate) (Minch Malts, Kildare, Ireland) was utilised during mashing. Malted barley had a moisture content of 4%, and a predicted spirt yield of >410 LA/tonne. Prior to use, the grains were also kept in cool, dark conditions. Grain was ground to each grain particle size—0.2, 0.65 and 1.1 mm—using a Buhler Miag disc (Buhler group, Londan, UK), and used immediately.

### 2.2. Predicted Spirit Yield

The predicted spirit yield was first determined on both wheat and malted barley samples, before commencing alcohol yield optimisations. This was carried out following the hot water extract method known as the European brewing convention (EBC) method 6.14 and carried out in triplicate [14]. Briefly, using a Buhler Miag disc mill (Buhler group UK), grain was ground to 0.2 mm, and 55 g of flour was weighed into previously heated stainless-steel beakers. A total of 360 mL of warmed water (65 °C) was added. The samples were mashed for 1 h at 65 °C, using mash baths (ICUBE s.r.o- R8, BS technologies, Yorkshire UK) in which the stainless-steel beakers are placed. This maintains the chosen temperature, while overhead stirrers mix the samples at 70 rpm. The saccharification rate was tested after 10 min, by taking a few drops of wort and adding a drop of iodine. This was repeated every 5 min until a yellow colour was obtained or until 1 h was complete. Post-mashing samples were cooled to 20 °C for 25 min, at which point the weight was adjusted to 450 g, using water preheated to 20 °C. Samples were filtered, using Whatman No. 1 paper, and the specific gravity was measured on an Anton Paar 5000 density meter (Anton Parr, Dublin, Ireland) The sample was transferred to a 500 mL Duran bottle, for fermentation, in which yeast (Pinnacle “M” type yeast (Ab Mauri, WHC labs, Wicklow, Ireland) was pitched at of rate of 0.4% (*w*/*w*). This was incubated at 30 °C for 72 h. Post-fermentation samples were filtered, and the final gravity was read on Anton Paar 5000 density meter. The predicted spirit yields were calculated using the following equations:

(1) OG or FG(°Sacch.)=1000−(SG−1)

where *SG* is the specific gravity of the wort of fermented filtrate at 20 °C.

*OG* is the original gravity and *FG* is the final gravity.

The soluble extract (*SE*) is calculated using the following:

(2) $$SE\left(\%\right)=\frac{OG\times 2.2279}{SG}$$

The % real fermentability (*F*) using the formula:

(3) $$F\left(real\;\%\right)=\frac{OG-FG}{OG}\times 100\times 0.814$$

The % fermentable extract (*FE*) using:

(4) $$Fermentable\;extract\left(\%\right)=\frac{SE\left(\%\right)\times F\left(\%\right)}{100}$$

Finally, the predicted spirit yield (*PSY*) using:

(5) $$PSY\;\left(LA.Tonne\right)=\frac{SE\left(\%\right)\times FE\left(\%\right)}{100}\times 6.06$$

The *PSY* was converted to dry weight basis using:

(6) $$PSY\;\left(LA.Tonne\;DWB\right)=\frac{PSY\times 100}{100-M}$$

where *M* is the moisture content as a percentage.

### 2.3. Alcohol Yield Analysis

The alcohol yields analysis method was based on that of Agu et al. [15], which stimulates the production process conditions in a “typical” Scotch whiskey grain distillery but was modified for a “typical” Irish grain whiskey distillery. The main differences relate to the use of enzymes, process temperature, and times. Briefly, wheat flour (5, 20 or 35 g) was obtained by milling the grains in a Buhler Miag disc mill, setting 0.2, 0.65, and 1.1 mm, was transferred into mashing beaker and slurred with water (86 mL preheated to 40 °C), with α-amylase (50 U/g of flour) (Sigma-Aldrich, Dublin, Ireland) and 100 mg/L calcium chloride (CaCl) (Sigma-Aldrich). The contents were gradually heated to 92 °C (temperature rise 2 °C/min), in a water bath, and cooked for 150 min. The cooked slurry was then cooled to 66 °C and given a second treatment of α-amylase (38 U/g of flour) and amyloglucosidase (0.22 U/g of flour) (Sigma-Aldrich). This was mashed then for 75 min, with a malt inclusion rate of 5% using high diastatic power distilling malted barley (cv. Laureate, Miag setting 0.2 mm). After cooling to 22 °C, the mash was pitched with distiller’s yeast (Pinnacle ‘M’ type) at a pitching rate of 0.4% (*w*/*w*) and adjusted to 250 g with water (20 °C). The mash was fermented for 72 h at 30 °C with the addition of 1.5 U/g of β-Glucanase (Sigma-Aldrich). The alcohol yield was determined from the alcohol strength of the distillate, which was measured using an Anton Paar 5000 density meter. The alcohol yield was quoted as litres of alcohol per tonne (LA/tonne) on a dry weight basis. All alcohol yield analyses were carried out in triplicate.

### 2.4. Experimental Procedure: Response Surface Methods

A response surface methodology (RSM) study, as described by Montgomery et al. [16], was conducted to determine the relative contributions of two dependent factors (grain amount (g) and grain particle size (mm)) to independent factor (alcohol yield). Values of each predictor factor were based on previous literature, detailed discussion with industrial stakeholders and professional judgement [7,8,17]. Grain amount was set from 5 to 35 g and grain particle size from 0.2 to 1.1 mm. A central composite design (CCD), with two-level full factorial with added centre and axial point, was used. It encompassed a face-centred cube with a triple replicated factorial (blocks on replicates were applied) and the centre point was constructed using the software package Minitab v.20.01 (Minitab, Dublin, Ireland). Maximum and minimum predicators were set following a review of current literature and considering professional advice. Three levels of each predictor were incorporated into the design. Table 1 shows the value range for each component and the combination of these levels used in the face-centred cube. Each experimental run was carried out in triplicate, when the RSM model taking this into account using blocks on reps. The response variable used to measure the optimum process was alcohol yield (LA/tonne). A multiple regression analysis of the data was carried out by surface response methodology and the second-order polynomial equation that defines predicted responses (*Y_i_*) in terms of the independent variables (*A* (grain flour (g)) and *B* (grain particle size (mm)):

(7) $$Y_{i}=b0_{i}+b1_{i}A+b2_{i}B+b11_{i}AA+b22_{i}BB+b12_{i}AB$$

where *Y_i_* = predicted response, *b*0*_i_* is the intercept term, *b*1*_i_* and *b*2*_i_* are linear coefficients, *b*11*_i_* and *b*22*_i_* are squared coefficients and *b*12*_i_* is an interaction coefficient. A combination of factors (*A* and *B*) represents an interaction between the individual factors in the respective term. These responses are a function of the level of factors. The response surface graphs indicate the effect of variables individually and in combination and determine their optimum levels.

### 2.5. Regression Analysis

Minitab was used to calculate regression equations from the response surface method data output. The regression equation was analysed according to the technique detailed above (Section 2.3). The accuracy of the regression equation was determined by varying the amount of grain and grain size, as indicated in Table 2, with all experimental runs being performed in triplicate. After determining alcohol yield, mean percentage errors were calculated, with a negative percentage error indicating an underperforming model and a positive percentage error suggesting a model that was overperforming. The mean percentage error (MPE) was calculated according to Equation (8).

(8) $$\mathrm{MPE}=\frac{100\%}{n}\sum_{i=1}^{n}\frac{{\mathrm{Alcohol}\;\mathrm{Yield}}_{\exp}-{\mathrm{Alcohol}\;\mathrm{Yield}}_{\mathrm{cal}}}{{\mathrm{Alcohol}\;\mathrm{Yield}}_{\exp}}$$

where alcohol yield_exp_ is the experimental value obtained during experiments and alcohol yield_cal_ was obtained from the regression equation.

Furthering this, regression equations were also used to depict the maximum grain amount during functional analysis. Briefly, theoretical alcohol yield was determined from 50 to 54 g using Equation (9). Studies were then conducted, in triplicate, using wheat grain ranging from 48 to 62 g (in increments of 2 g) at a particle size of 0.2 mm, following the process described in Section 2.3. The purpose of this was to determine the actual alcohol yield, in order to determine the maximum amount of grain that can be added.

### 2.6. Comparison of Grain Flour Amounts

A follow-up experiment was conducted to see if there was a significant variation in alcohol yield when utilising 25, 30, or 35 g of wheat flour with a 0.2 mm particle size. The null hypothesis that there is no significant difference in alcohol yield between the three grain amounts was tested using a one-way ANOVA in Minitab. The procedure described for alcohol yield analysis (Section 2.3) was used to conduct these tests. All of the samples were run in triplicate.

## 3. Results and Discussion

### 3.1. Predicted Spirit Yield

The EBC standard method was used to determine the predicted spirit yield [14]. Because no additional enzymes or chemical compounds are introduced, this approach is better suited to malted grains. Unmalted grains lack the natural enzymes present after malting, necessitating the use of enzymes, malted barley, and synthetic substances such as calcium to obtain maximum alcohol yields [12]. Furthermore, high-temperature cooking of grains is used to gelatinise the starch, which aids in the process of breaking down cell walls on the ground flour [12,18]. Costello had a predicted spirit yield of 171.6 LA/tonne. When compared to the alcohol yield reached in jurisdictions outside Ireland, the yield obtained is poor. The predicted spirit yield of typical distilling wheat is <450 LA/tonne dwb [8,17]. Soft endosperm wheat is also preferred due to its low protein and high starch content. Hard wheat has a denser endosperm and a higher gluten content, making it more difficult to extract the starch. Alongside this, protein in hard wheat typically holds a strong bond to starch, and coats protein adheres to the starch surface in a strong matrix [19]. Additionally, the increased protein content of hard wheat appears to cling to the starch, preventing enzymatic breakdown even after cooking [12,19,20,21]. Costello, hard wheat that is commonly cultivated in Ireland, is usually utilised as livestock feed, and few researchers have looked at its potential for usage in spirit production. Hard wheat is favoured in Ireland due to its high yields, disease resistance, and suitability in the Irish climate [22,23].

### 3.2. Alcohol Yield from Costello

**Figure 1:**
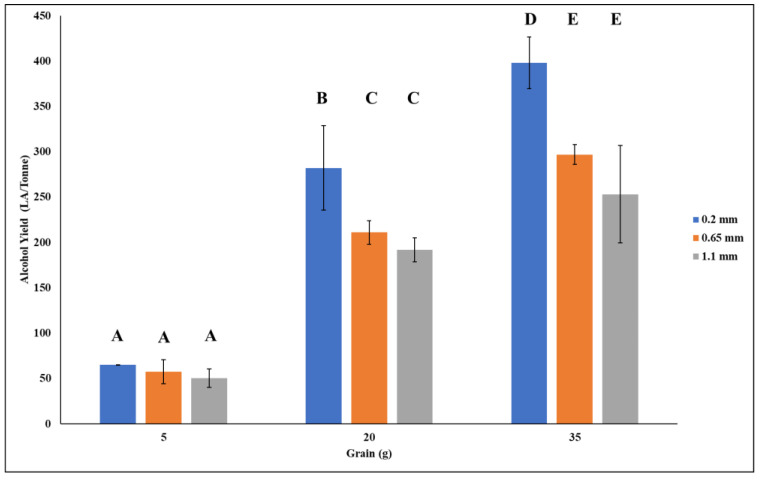
Mean alcohol yield achieved using different grain amounts and grain particle sizes. Results are presented as mean (*n* = 3) and error bars indicating standard deviation. The same letter indicated no significant differences, as determine by one-way ANOVA with the Tukey post hoc test.

The alcohol yield obtained in this study varied based on grain particle size and the amount of grain used. As expected, grain amount had the greatest impact on alcohol yield. Thinner mashes, according to the literature, give lower alcohol yield than thicker mashes [24,25,26]. The smallest grain amounts used in the trials was 5 g, resulting in a grain to liquid ratio of 17.2. Due to a paucity of grist, this grain size was not predicted to attain a high alcohol yield across each grain particle size. Alcohol yields ranged from 50 to 70 LA/tonne dry weight (Figure 1). A one-way ANOVA was used to determine whether grain particle size had any effect on alcohol yield using 5 g of flour, and there were no significant differences found (*p* = 0.376). It was expected that as the grain amount increased so would alcohol yield. A mean alcohol yield of 191–282 LA/tonne was obtained using 20 g of wheat (grain to liquid ratio 4.3) (Figure 1). There were no significant differences in alcohol yield across grain particle sizes, just as there were no significant differences in grain particle size over 5 g (*p*-value 0.053). The largest grain particle size investigated, 35 g of flour (grain to liquid ratio 2.45), yielded the highest yields across all grain particle sizes, with an average alcohol yield of 253–398 LA/tonne. However, significant disparities in alcohol yield were detected across each grain particle size at this grain size (*p*-value 0.017). There was no discernible change in yields between 0.65 and 1.1 mm. When compared to 0.65 and 1.1 mm, the Tukey post hoc tests revealed a significant difference in 0.2 mm grain particle size. Figure 1 shows that 0.2 mm grain particle size at 35 g of flour results in the highest alcohol yields.

Limited research on the impact of grinding parameters on the total alcohol yield process has been published for alcohol yield manufacturing [7]. Because there was such a wide range of results when using varied grain amounts and grain particle sizes, additional research and analyses were required to establish if there was a link between the variables. This, as the first step, may be one of the most critical. According to research on biofuels and biogas, the amount of biomass used at the start as well as the grain particle size have a significant impact on the yields obtained [27,28]. A study conducted by Moeller et al. [28] using Triticale grain kernels for biogas production indicated that smaller grain particle sizes gave higher biogas yields compared to larger grain particle sizes. Moreover, a report carried out by Smith et al. [7] noted that the finesses of milling can impact alcohol yields, with finely ground meal yielding 5–10% more alcohol than a coarser ground meal. The report also notes that it is assumed that due to flour undergoing cooking and gelatinisation steps, the finest of grinding is less important. However, as shown in this study, results varied across different particle sizes, though it is clear that grain particle size has an immense impact on the potential alcohol yield. For example, at 35 g of grain, there is a difference in yield of 100 LA/tonne observed between 0.2 and 0.65 mm particle sizes (Figure 1), which further increases to a difference of 145 LA/tonne when comparing 0.2 and 1.1 mm grain particle sizes. Further work is now needed to determine starch digestibility across different grain particle sizes and its conversion to fermentable sugars.

Grain amounts also play a vital role in alcohol yield. The more grain that is added, the higher the alcohol yield. However, it is important to note that as grain amount increases, it would be expected that alcohol yield would plateau. This is due to numerous reasons such as the thickness of the mash, causing processability concerns, such as inadequate mixing and absorption of water. It is important to note that thicker mashes are preferred over thinner mashes due to the protection they offer in terms of enzyme inactivation. It has been noted by Saarni et al. [25] that the thermostability of the key amylolytic enzymes increases with mash thickness, such that, for particularly high temperatures, thicker mashes yield more fermentable worts. As such, it is important to investigate the ratio of grain to liquor that will yield high fermentable worts. This study showed that 35 g of hard wheat grain yields a high alcohol level, in the region of 390 LA/tonne, while lower amounts of grain, 5 and 20 g, yielded significantly less alcohol (*p* = 0.185) (Figure 1). To fully optimise a process and attain the highest yield of alcohol, all process elements must be given equal weight; this is the primary objective of optimising these parameters using the response surface method.

### 3.3. Response Surface Methodologies—Alcohol Yield

Response surface methods use a design of experiments approach to discover the optimal parameter for grain amount and grain particle size in the production of spirit during the lab-scale operation. This approach of conducting experiments allows for the detection of significant differences between variables, the calculation of optimal parameters, and the investigation of dependent factor interactions. The response surface method model identifies statistically significant and functional correlations while looking at the potential alcohol yield.

#### 3.3.1. Statistical Significances

**Figure 2:**
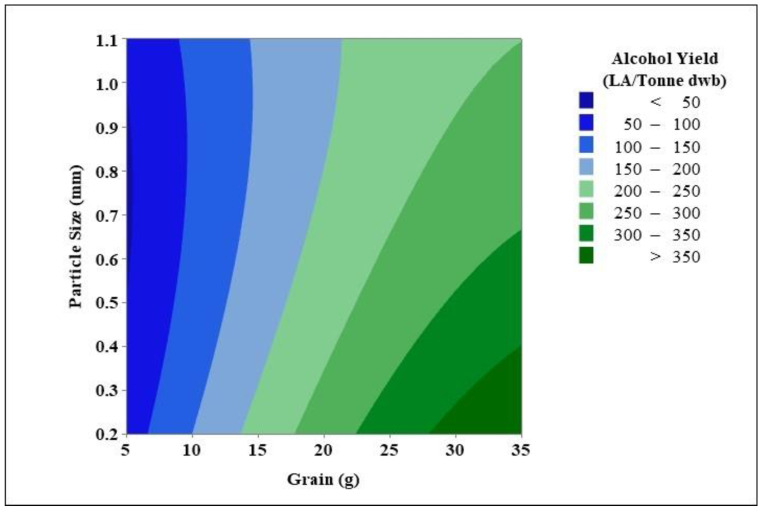
Contour plots showing the mean alcohol yields achieved during the response surface model, using varying grain amounts and grain particle sizes.

The statistical significance of the model is determined using analyses of variance (ANOVA). The null hypothesis of no link between the dependent (grain amount and grain particle size) and independent variables can be rejected because the entire model *p*-value (4.6 × 10^−15^) is less than the level of significance (0.05), as shown in Table 3. As a consequence, both dependent factors have an impact on the alcohol yield obtained. The amount of alcohol produced is affected by both the linear and quadratic (square) variables. When each term is considered as a dependent variable, they have a distinct linear effect on alcohol yield, while when consider together or with the power of each variable combined, they show a polynomial graph, indicating a maximum alcohol yield will be achieved. The ANOVA *p*-values (Table 3) yielded results that were similar to those mentioned in alcohol yield from Costello. The linear terms are concerned with the effect of each variable on alcohol yield. Both variables have a *p*-value > 0.05, indicating that there are significant differences in the outcomes. This can also be seen for each variable’s squared interaction. The *p*-value for grain quantity is 0.001, showing that there is a significant variation in yields, whereas the *p*-value for grain particle size is 0.053, indicating that there is no significant difference in alcohol yield across each milling size. Furthermore, the interaction of both dependent variables yields a *p*-value of 0.001 that is lower than the significance level, showing that the interaction of these two variables affects alcohol yield. This showed that the combined power of these two variables has a considerable effect on alcohol yield. Moreover, Figure 2 shows that at a 0.2–0.4 mm grain particle size and using 15 g of grain, the mean alcohol yield is between 150 and 200 LA/tonne; but at the same grain particle sizes and 35 g of grain, the mean alcohol yield is >350 LA/tonne. Finally, the model exhibits no lack of fit because the *p*-value is greater than the level of significance (Table 3).

**Figure 3:**
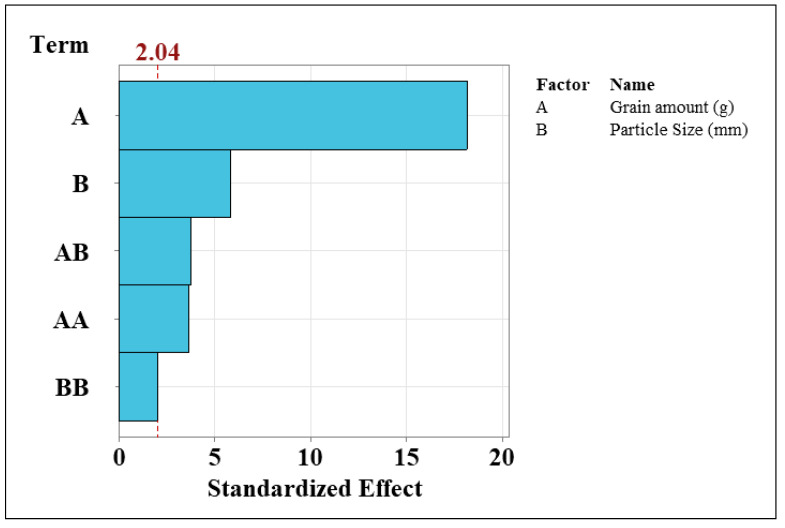
Pareto charts examining the standardised effects of the dependent variable (alcohol yield) on the independent variable (grain amount and size). All dependent variables above the 2.04 line indicated significant differences, meaning they have an important impact on the alcohol yield achieved. Furthering this, each variable is listed in order of importance to the model.

**Figure 4:**
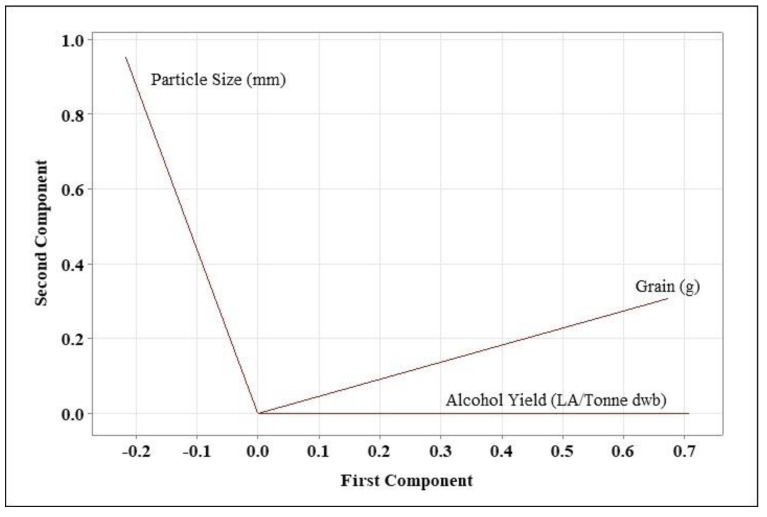
Principal component analysis of the dependent (alcohol yield) and independent variables (grain amount and particle size) used in this study. The amount of grain used has the biggest impact on AY, due to the closeness of the lines for alcohol yield and grain amount.

The normalised impacts of both the dependent and independent variables in response to the independent variable are shown in Figure 3. The terms are arranged in ascending order of importance. Figure 3 shows that A (quantity of grain) has a higher impact on the system than B (grain particle size). Moreover, grain particle size and the interaction of A and B has a significant impact on the process. When looking at grain flour quantities, grain particle size and alcohol yield, principal component analysis reveals a stronger link between alcohol yield and grain flour quantities (Figure 4). These findings imply that grain flour quantity is a controlling factor, and that alcohol yield will increase only because of this. The size of grain particles is crucial, yet it has little impact on alcohol yield. This was noted previously, in that there were no significant differences across particle sizes for 5 and 20 g of flour, while at 35 g a significant difference was seen between grain particle sizes, with a grain particle size of 0.2 mm yields, resulting in the highest alcohol yield.

According to the adjusted R-square value of 90.67% (Table 4), grain particle size and grain amount explain variance in the alcohol yield obtained, indicating that the model has good practical importance. Finally, a multicollinearity test called the variance inflation factor revealed that the dependent variables are unrelated (Table 3). While each of these factors has an impact on alcohol yield on its own, the response surface method model indicates that they work together to influence yields. All of this indicated that the data were in good agreement with the response surface method model.

#### 3.3.2. Functional Relationships

The functional link between each variable’s impact on alcohol yield was explored to see how each parameter’s independent and combined effects on alcohol yield interacted with one another. This was achieved through the use of factorial plots and main effects plots, as well as the regression equation produced during the response surface method data analysis.

**Figure 5:**
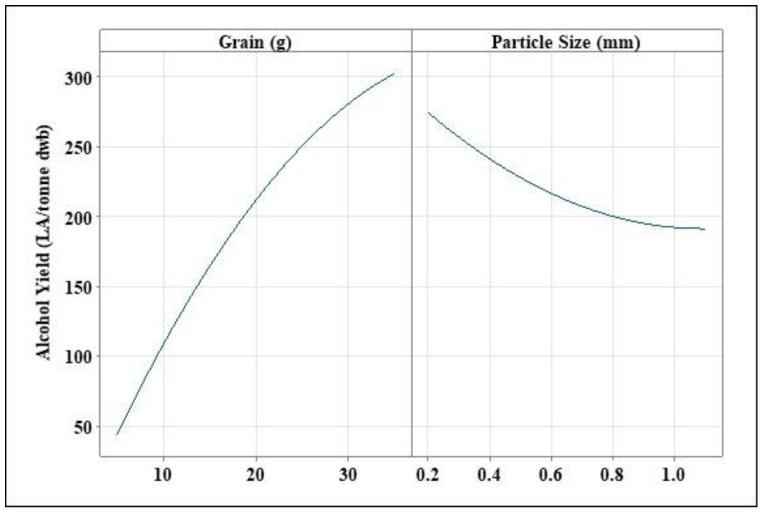
Main effect plot for both independent variables used within this study. Gain amounts show a positive polynomial graph, showing alcohol yield increases as grain amount increases, while alcohol yield is shown to decrease as grain particle size increases at a linear rate.

**Figure 6:**
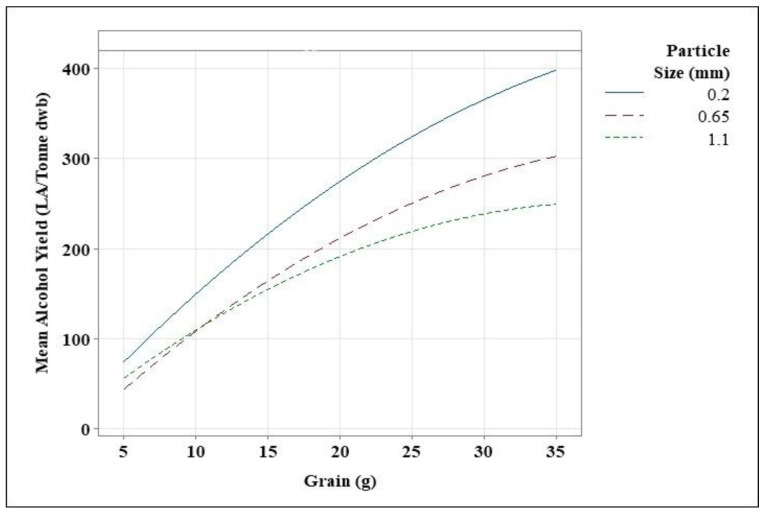
Interaction plots display the effect that both grain amount and grain particle size have on alcohol during the response surface model. This graph shows that a grain particle size of 0.2 mm at 35 g of grain will produce the highest yield.

The factorial plot of the dependent variables was examined (Figure 5 and Figure 6). The main effect plot (Figure 5) shows how each variable affects alcohol yield. It is clear that as grain quantity increases, so does alcohol yield and that by extrapolation, this graph indicates a polynomial (quadratic) equation (regression equation and predictive analysis). This indicates that it is likely to reach a maximum alcohol yield in terms of grain quantities before gradually dropping or plateauing. Either outcome would be expected for a number of reasons. The first relates to processability issues. While thicker mashes are known to provide higher yields, when the grain to liquid ratio is too high, a cooked cake forms [12,25,29]. This means that grain flour absorbs a lot of water, leaving relatively little liquid and making it difficult to blend [19,22]. If starch is not gelatinised successfully, the conversion to sugars will be limited [7,12,30]. In thick mashes, insufficient mixing may cause a pocket of dry flour, meaning that starch could not be gelatinised successfully, in which case the conversion to sugars would be limited [12]. Furthermore, it is known that hard wheat has a higher water absorption capacity [22]. Therefore, hard wheat is considered as a challenge from a processability perspective. One way to overcome this is to increase the initial water added to make wort. However, this generates a thinner mash, increases energy cost for cooling and breaks away from industry norms [12,18]. Moreover, the thickness of the mash affects the activity and efficiency of enzymes [25,26,31]. The fraction of enzymes present in thicker mashes is higher, and so they are more concentrated. They are also more resistant to the denaturing effects of high temperatures, allowing them to work faster and for longer periods [25,26]. However, too thick of a mash also has a negative impact, as previously discussed.

The size of the particles has a significant impact on alcohol yield; as observed in Figure 5, alcohol yield decreases as grain particle size increases. Grain particle size has a negative linear slope. Finer flours, especially of hard wheat, make the starch more accessible, resulting in more reducing sugars being produced. Over 50 LA/tonne is lost as the grain particle size is increased from 0.2 to 1.1 mm (Figure 6), a significant drop in alcohol yield.

Interaction plots were used to examine how the dependent and independent factors interact with the independent variable and Figure 6 depicts the results of some interesting observations. The grain particle size of 0.2 mm consistently produces a higher alcohol yield than either 0.65 or 1.1 mm in all grain amounts. Alcohol yield at 35 g grain ranges from 300 to 400 LA/tonne, depending on grain particle size. Optimum yields were found at 0.2 mm grain size, which produces one-third more alcohol than either 0.65 or 1.1 mm. This demonstrates that a grain size of 0.2 mm produces the greatest results and is therefore considered the ideal grain particle size. In terms of alcohol yield, there are several interesting findings between 0.65 mm and 1.1 mm grain sizes. Initially, 1.1 mm gave significant alcohol yields at 5 g of grain compared to a grain particle size of 0.65 mm (*p* value = 0.025), but as grain quantities increased to 10 g, both 0.65 and 1.1 mm yielded the same quantity of alcohol. When grain flour quantities increased further (35 g), alcohol yield was greater at 0.65 mm (300 LA/tonne) than at 1.1 mm of grains (approximately 253 LA/tonne) (*p* = 0.0005). The grain size of 1.1 mm, and the combination of hard wheat adsorbing water meant that there was inadequate mixing. This was noted during both cooking, where a thick cake formed, and during mashing, when the malted barley that was added remained on the top of the cake rather than being homogenous with the wort. This is, however, something that could be corrected during cooking if a lower temperature was used. Furthermore, it was noted that at lower cooking temperatures during experimental runs, less of a cake formed at 72 and 82 °C, when compared against 92 °C. Equally, liquefaction of starch seems to occur at a quicker rate at lower temperatures. However, this could also be attributed to the α-amylase working more efficiently at a lower temperature [12,30,32]. Nevertheless, a lower temperature may mean that starch is not fully gelatinised, or it could be retrograded back, meaning a limited amount of starch to be converted to fermentable sugars [12,30].

The functional relationship between the dependent variable and the independent variables was also examined using regression equations. In this scenario, a regression equation was created using response surface method analysis, which considers the impact of each variable on the final alcohol yield. The regression equation considers each variable’s linear, quadratic, and interaction properties. The regression equation is represented as Equation (9) below. The regression equation determines alcohol yield based on a dry weight basis, as a dry weight basis was used to analyse and input all alcohol yield into the data set.

(9) AY=12.9+18.59 (A)−132.6 (B)−0.1709 (AA)+105.1 (BB)−4.83 (AB)

where *A* is grain flour quantity (g), and *B* is grain particle size (mm)

The linear terms have both a positive and a negative impact on alcohol yield, as can be seen in Equation (9). For example, while each gram of grain added to the process yields an extra 18.59 LA/tonne, as grain particle size increases, alcohol yield production also increases. This indicates that greater alcohol yield is achieved when grain amounts are increased and when lowering grain particle size. Grain amounts are a negative element in the quadratic equation, suggesting that there is a maximum amount of grain that can be added to provide optimal yields, after which alcohol yield decreases. At between 50 and 54 g of grain (Equation (9)), the maximum alcohol yield is theoretically obtained based on the regression equation, after which the yield should drop. Grain amounts ranging from 48 to 62 g were studied at a grain particle size of 0.2 mm. It would be expected that theoretical yields of 442–445 LA/tonne would be achieved between 50 and 54 g. However, this was not the case. Maximum yield was in fact obtained at 48 g, achieving 403.50 LA/tonne, while 50–54 g only achieved 315–325 LA/tonne. Alcohol yield at 48 g, 403.50 LA/tonne, is greater than alcohol yield (398.25 LA/tonne) achieved at 35 g, but marginally. One-way ANOVA showed no significant difference in alcohol yield achieved using 35 and 48 g of grain (*p*-value = 0.321); therefore, adding more grain to achieve slightly higher alcohol is futile.

It was observed during cooking that grain swelled, and water absorption causes clumping of flour and a thick paste. When malted barley was added during mashing, inadequate mixing was observed in samples containing 50–62 g of grains. Alcohol yield ranged from 282 to 325 LA/tonne when grain amounts varied from 50 to 62 g, with 62 g producing the least amount of alcohol. This was to be expected due to the processability issues observed when using 35 g of grain at each grain particle size. Even though the equation indicated maximum yields at 50–54 g of grain, the processability issue can have a drastic impact on the alcohol yield achieved. Despite the fact that grain particle size has a positive impact on the regression equation (+105.1), alcohol yield decreases as grain particle size increases. Finally, the interaction of grain quantity and grain particle size is viewed as having a negative impact on the system. Nevertheless, this coefficient considers both grain quantity and grain particle size, implying that the higher the grain and the smaller the grain particle size, the greater the impact on alcohol yield.

#### 3.3.3. Overall Alcohol Yield

Various alcohol yields were obtained over the course of this study. The amount of grain flour utilised, as well as the grain particle size, influenced alcohol yield. This study aimed to determine the greatest grain particle size and amount of grain flour to maximise alcohol yield. The response surface approach is a technique for determining the best dependent variables for a given independent variable. Contour plots can be made by using this method (Figure 2). This graph depicts the relationship between alcohol yield and the amount of grain with respect to grain particle size. The predicted spirit yield of Costello was calculated at 171.6 LA/tonne. Alcohol yield achieved during this study was equal to the predicted spirit yield by utilising 10–17 g of grain with grain particle sizes ranging from 0.2 to 1.1 mm, after which, as grain amount increased, the alcohol yield surpassed the predicted spirit yield. Alcohol yield increased as grain quantity increased, and it increased further as grain particle size dropped. The best yields were obtained by utilising 30–35 g of grain with a grain particle size of 0.2–0.35 mm. Alcohol yields were greater than 350 LA/tonne within this range but remained below 400 LA/tonne. Based on the adjustment of milling parameters, the ideal range for most alcohol is between 0.2 and 0.3 mm grain particle size and 30–35 g flour. Because 30–35 g of flour provides similar alcohol yields, further research is needed to determine if there are any major changes.

The alcohol yields obtained during this study were lower than those reported in the literature [7,18,33,34,35,36]. Currently, investigations on alcohol yield follow a process design for the manufacturing of Scotch whiskey. These studies cover a wide range of wheat varieties, including hard and soft endosperm, as well as varied environmental conditions such as nitrogen fertilizer rates. Kindered et al. [36] looked at alcohol yield from hard wheat, cv. Option, and found that it had an alcohol yield of 439–463 LA/tonne, depending on the N rates used. The yields observed were lower in this study, but the process conditions were different in that the initial cooking temperature was lower (92 °C), whereas in papers published by Scottish Whiskey Research Institute (SWRI), flour was cooked at 142 °C. Furthermore, when employing soft wheat varieties such as Riband, Viscount, Claire, and Consort, alcohol yield in other tests varied from 410 to 460 LA/tonne. These were all cooked at the same temperature of 142 °C [17,18,22,23,24,25,34,36]. Green et al. [18] looked at soft wheat Viscount with a cooking temperature of 85 °C and 30 g of flour with a grain particle size of 0.2 mm. The researchers also used α-amylase in their trial, but they used a 20% malt inclusion rate instead of 5%, as reported in this study. They reported an alcohol yield of 450–460 LA/tonne. Comparing alcohol yield based on different process parameters is futile, despite the increased yields. While the focus of this study is on milling parameter optimisation, both the cooking and mashing stages require improvement. During these procedures, the temperature of cooking and mashing, as well as the type and amount of enzymes utilised, must all be optimised. The alcohol yield is projected to rise once all relevant parameters have been optimised. Further research is needed to determine if there are any major changes in alcohol yield between 30 and 35 g of grain flour, as they both produce similar alcohol yields. Further optimisation of each stage is required to achieve a successful method for testing small quantities of grain, based on Irish whiskey process parameters.

#### 3.3.4. Predictive Analysis

The regression equation (RE) was determined from the response surface method model; while this equation can be used to determine potential theoretical alcohol yield (alcohol yield_cal_) when both grain particle size and amount are altered, all theoretical values need to be confirmed experimentally. During these studies, the regression equation was determined from the response surface method model and displayed as Equation (9). From Equation (9), alcohol yield based on dry weight can be determined from Equation (6), by substituting alcohol yield for predicted spirit yield. During this experiment, random values were substituted into Equation (9) (Table 2), and this experimental parameter was used in determining alcohol yield. Seven different combinations of variables were examined. The grain size of 1.1 mm showed the largest deviation between alcohol yield_exp_ and alcohol yield_cal_. The average MPE for 1.1 mm was −13.83, meaning that the experimental value is underperforming when compared to the value calculated based on the regression equation. There are numerous reasons why such a large deviation is observed. Regression equations do not take into account processability issues during experimental runs. It was noticed that as grain size 1.1 mm was heated at 92 °C, the grain swelled as expected, absorbing the majority of liquor present, indicated a lack of mixing, and forming a thick cake. This, however, was mainly seen in larger grain amounts (20 and 30 g). As the grain size decreased, the MPE value dropped closer to zero. The average MPE for 0.2 mm grain size was −1.69, and −5.6 for 0.65 mm. This indicated that these runs are performing closer to the value obtained from the regression equation, giving a better fit to the model, showing that the MPE can assist in making informed decisions when calculating theoretical yields from this procedure. For instance, if 50 g of flour with a grain particle size of 0.2 mm is used, the alcohol yield_cal_ is 444 LA/tonne. However, because the experimental run is underperforming by 1.6%, the true alcohol yield should be 432.6 LA/tonne. Having the ability to make this calculation allows for a judgement to be made before running the process, potentially offering significant cost savings to producers.

### 3.4. Investigation of Optimum Grain Amount

According to the response surface method model, grain levels of 30 and 35 g yielded above 350 LA/tonne. Despite the fact that 35 g of grain flour is recommended by the response surface method model, several studies have revealed that 30 g of grain flour is adequate [8,16,33]. As a result, a follow-up experiment was designed to investigate the alcohol yield from varied grain amounts (Section 2.6). The purpose was to evaluate if grain amount had changed significantly. Table 5 shows the alcohol yield collected during these studies. The difference between 25 g (324.5 LA/tonne) and both 30 g (389.5 LA/tonne) and 35 g (398.25 LA/tonne) is apparent (Table 5). One-way ANOVA determined that there is a significant difference in the sample set (*p* < 0.05), while the Tukey post hoc test indicates where the significant differences lie. Within Table 5, a significant difference is indicated by a different letter. Between 30 and 35 g grain, there is no discernible change. The average difference is approximately 9 LA/tonne of grain. This is minor in a large-scale setup, but it is worth noting that using a larger amount of grain can cause processability concerns (thicker mash). As the grain swelled during the cooking stage, less liquid was present, resulting in a lumpy grist as samples were mashed. In addition, the liquefaction of 35 g samples took longer to begin (90 min) than the liquefaction of 30 g samples, which took 60 min. Furthermore, it was observed that when malted barley was introduced during mashing, it took longer for the barley to homogenise inside the samples at 35 g than it did at 30 g. Finally, the use of 30 g of wheat flour is comparable to published research in which wheat is examined for its potential to be used or for the generation of spirit. Papers published by Agu et al. [8,15], Smith et al. [7,17] and Kindred et al. [35,36], all used 30 g of grain. As a result of the lack of meaningful differences, 30 g of wheat is recommended in experiments with an optimised grain particle size of 0.2 mm. Furthermore, the cost-saving impact of producing alcohol with 30 g of grain and reducing processability issues is of benefit to the industry.

## 4. Conclusions

The primary goal of this research was to determine the most effective parameter for milling grain in order to obtain the maximum potential alcohol concentration produced. Costello, hard wheat, was used in the trials, and the RSM technique was applied. The key findings show that as grain particle size increases, alcohol yield decreases, with 35 g of grain at a 0.2 mm particle size yielding one-third more alcohol than other particle sizes in this study. Optimum yields were achieved using 30 g of grain at a particle size of 0.2 mm with limited processability issues; therefore, it is recommended going forward that these milling parameters should be used. Further, the response surface model is suitable for use, but overpredicted alcohol yield by ignoring processability concerns. It is now recommended that to establish a complete method for assessing small batches of Irish wheat that mimics industrial production norms, further work is necessary to optimise liquefaction and saccharification in the Irish whiskey production process. Furthering this to develop a well-rounded standard technique, more wheat samples, such as soft wheat and different varieties, should also be studied.

## Figures and Tables

**Table 1:** Experimental ranges of the two variables studied, grain flour and particle size, using response surface methods, a central composite design in terms of actual and coded variables, alongside the experimental runs that were carried out during the trial, which were conducted in triplicate.

Variables	Symbol	Coded Levels		
		Low (−1)	Mid (0)	High (1)
**Grain flour (g)**	**A**	5	20	35
**Particle size (mm)**	**B**	0.2	0.65	1.1
**Experimental runs**	**Coded**		**Uncoded**	
	**A**	**B**	**A**	**B**
1	1	0	35	0.65
2	0	1	20	1.1
3	0	0	20	0.65
4	0	−1	20	0.2
5	0	0	20	0.65
6	0	0	20	0.65
7	−1	0	5	0.65
8	1	−1	35	0.2
9	−1	1	5	1.1
10	0	0	20	0.65
11	0	0	20	0.65
12	1	1	35	1.1
13	−1	−1	5	0.2
14	0	0	20	0.65

**Table 2:** Regression equation experimental plan based on grain amount and size (Section 2.5), the average experimental alcohol yield (AY_exp_) achieved during experimental trial and the predicted alcohol yield (AY_cal_) from the regression equation and the MPE used to check the accuracy of the regression equation.

Sample ID	Amount of Grain (g)	Size of Grain	AYexp (LA/Tonne DW)	AYcal (LA/Tonne DW)	MPE (%)	Average MPE
RE1	10	0.2	149.15	149.75	−0.40	
RE2	20	0.2	262.5	274.73	−4.45	
RE3	30	0.2	364.67	365.55	−0.24	−1.69
RE4	10	0.65	106.46	108.55	−1.92	
RE5	20	0.65	192.15	211.79	−9.27	−5.56
RE6	10	1.1	95.95	109.92	−12.70	
RE7	20	1.1	203.03	238.74	−14.95	−13.83

Where AYexp relates to the experimental alcohol yield achieved during this study; AYcal relates to the theoretical alcohol yield, as predicted using the regression equations.

**Table 3:** Analysis of variance (ANOVA) for alcohol yield from response surface methodologies analysis based on grain amount and size. This table indicates both *p*-value and VIF.

Source	DF	F-Value	*p*-Value	VIF
**Model**	10	40.86	0.00	
**Blocks**	5	3.26	0.02	
**Linear**	2	181.89	0.00	
Amount of Grain (g)	1	329.53	0.00	1
Grain Size (mm)	1	34.24	0.00	1
**Square**	2	6.9	0.00	
Amount of Grain (g)*Amount of Grain (g)	1	13.26	0.00	1.26
Grain Size (mm)*Grain Size (mm)	1	4.06	0.05	1.26
**2-Way Interaction**	1	13.99	0.00	
Amount of Grain (g)*Grain Size (mm)	1	13.99	0.00	1
**Error**	31			
Lack of Fit	19	1.56	0.22	
Pure Error	12			
**Total**	41			

Where, VIF is the Variance inflation factors, the F value is the **F distribution** and DF is the degrees of freedom.

**Table 4:** Model summary from RSM analysis, displaying the R-squared and adjusted R-squared values.

Factor	S	R-sq	R-sq(adj)	PRESS	R-sq(pred)
Alcohol yield	0.362451	92.95%	90.67%	8.06019	86.04%

Where S represents the standard deviation of the distance between the data values and the fitted values. S is measured in the units of the response. R-sq is the percentage of variation in the response that is explained by the model. R-sq(adj) is percentage of the variation in the response that is explained by the model, adjusted for the number of predictors in the model relative to the number of observations. PRESS is the prediction error sum of squares (PRESS), a measure of the deviation between the fitted values and the observed values and R-sq(pred) is the predicated R-sq value, which indicates how well a model without each observation would predict that observation.

**Table 5:** Average alcohol yield achieved during experiments to test if there was a significant difference between using 25–35 g of grain flour. All results are the mean ± standard deviation (*n* = 3). Different letters indicate a significant difference.

Grain Flour (g)	Particle Size (mm)	AY (LA/Tonne)	
25	0.2	324.5 ± 8.5	A
30	0.2	389.5 ± 5.5	B
35	0.2	398.25 ± 6.4	B

## Data Availability

The data sets generated for this study are available on request to the corresponding author. Data has been stored in the library repository of Institute of technology Carlow, Ireland.
